# Effects of the lockdown on the mental health of the general population during the COVID-19 pandemic in Italy: Results from the COMET collaborative network

**DOI:** 10.1192/j.eurpsy.2020.89

**Published:** 2020-09-28

**Authors:** Andrea Fiorillo, Gaia Sampogna, Vincenzo Giallonardo, Valeria Del Vecchio, Mario Luciano, Umberto Albert, Claudia Carmassi, Giuseppe Carrà, Francesca Cirulli, Bernardo Dell’Osso, Maria Giulia Nanni, Maurizio Pompili, Gabriele Sani, Alfonso Tortorella, Umberto Volpe

**Affiliations:** 1 Department of Psychiatry, University of Campania “L. Vanvitelli”, Naples, Italy; 2 Department of Medicine, Surgery and Health Sciences, University of Trieste and Department of Mental Health, Azienda Sanitaria Universitaria Giuliano Isontina – ASUGI, Trieste, Italy; 3 Department of Clinical and Experimental Medicine, University of Pisa, Pisa, Italy; 4 Department of Medicine and Surgery, University of Milan Bicocca, Milan, Italy; 5 Center for Behavioral Sciences and Mental Health, National Institute of Health, Rome, Italy; 6 Department of Biomedical and Clinical Sciences Luigi Sacco and Aldo Ravelli Center for Neurotechnology and Brain Therapeutic, University of Milan, Milano, Italy; 7 Institute of Psychiatry, Department of Biomedical and Specialty Surgical Sciences, University of Ferrara, Ferrara, Italy; 8 Department of Neurosciences, Mental Health and Sensory Organs, Faculty of Medicine and Psychology, Sapienza University of Rome, Rome, Italy; 9 Department of Neuroscience, Section of Psychiatry, University Cattolica del Sacro Cuore, Rome, Italy; 10 Department of Psychiatry, Fondazione Policlinico Agostino Gemelli IRCCS, Rome, Italy; 11 Department of Psychiatry, University of Perugia, Perugia, Italy; 12 Clinical Psychiatry Unit, Department of Clinical Neurosciences, Università Politecnica delle Marche, Ancona, Italy

**Keywords:** Anxiety, COVID-19, depression, lockdown, pandemic, stress

## Abstract

**Background:**

The Coronavirus disease 2019 (COVID-19) pandemic is an unprecedented traumatic event influencing the healthcare, economic, and social welfare systems worldwide. In order to slow the infection rates, lockdown has been implemented almost everywhere. Italy, one of the countries most severely affected, entered the “lockdown” on March 8, 2020.

**Methods:**

The COvid Mental hEalth Trial (COMET) network includes 10 Italian university sites and the National Institute of Health. The whole study has three different phases. The first phase includes an online survey conducted between March and May 2020 in the Italian population. Recruitment took place through email invitation letters, social media, mailing lists of universities, national medical associations, and associations of stakeholders (e.g., associations of users/carers). In order to evaluate the impact of lockdown on depressive, anxiety and stress symptoms, multivariate linear regression models were performed, weighted for the propensity score.

**Results:**

The final sample consisted of 20,720 participants. Among them, 12.4% of respondents (*N* = 2,555) reported severe or extremely severe levels of depressive symptoms, 17.6% (*N* = 3,627) of anxiety symptoms and 41.6% (*N* = 8,619) reported to feel at least moderately stressed by the situation at the DASS-21.

According to the multivariate regression models, the depressive, anxiety and stress symptoms significantly worsened from the week April 9–15 to the week April 30 to May 4 (*p* < 0.0001). Moreover, female respondents and people with pre-existing mental health problems were at higher risk of developing severe depression and anxiety symptoms (*p* < 0.0001).

**Conclusions:**

Although physical isolation and lockdown represent essential public health measures for containing the spread of the COVID-19 pandemic, they are a serious threat for mental health and well-being of the general population. As an integral part of COVID-19 response, mental health needs should be addressed.

## Background

There is no doubt that the Coronavirus disease 2019 (COVID-19) pandemic, and its related containment measures such as lockdown, is affecting mental health of the general population worldwide [1–3]. This is an unprecedented event, which is influencing the healthcare, political, economic, and social systems [4]. Given the high level of contagiousness, as well as the lack of appropriate treatments and vaccines, almost all countries have adopted confinement measures, including lockdown, home isolation and physical distancing [5]. While most of the clinical and research efforts have been directed to reduce the effects of the virus on physical health [6–8], its short- and long-term effects on mental health are causing a second wave of pandemic, which has been mostly neglected [9–11]. Furthermore, the pandemic represents a traumatic event which has differential effects at individual and population levels. At the individual level, high rates of depression, anxiety, fear, panic, anger, and insomnia have been documented in studies mainly carried out in China or from short-term reports [12–14]. At the population level, the pandemic is associated with a range of psychosocial adversities, including economic hardship and financial losses (due to unemployment and reduced income), school closures, inadequate resources for medical response, domestic violence, and deficient distribution of basic good necessities [15]. The psychopathological consequences include the fear of contracting the disease and of dying, losing livelihoods and loved ones, uncertainty and worries about the future, social discrimination, and separation from families and caregivers [16–19]. This is why the current pandemic represents a new, complex and multifaceted form of psychosocial stressor [20], being completely different from other natural disasters [21], such as earthquakes or tsunamis [22, 23], wars, terroristic attacks, mass conflicts, or economic crisis [24–31], and also from previous epidemics, such as severe acute respiratory syndrome (SARS), Middle East respiratory syndrome (MERS) and Ebola [32, 33].

Italy has been the first western country heavily affected by the pandemic, and it has been the country with the highest number of infected and dead people for many weeks [34]. On March 8, 2020, the Italian Prime Minister has placed 60 million people under lockdown. This measure has been prolonged for 8 weeks, until May 3, 2020. This period is known as “Phase one,” during which all not necessary activities have been closed, more than 29,000 people have died and almost 100,000 people have been home-isolated. During the initial phase of the pandemic, the outbreak in Italy seemed to have a greater severity of the disease, with a higher case fatality rate (CFR) than previously observed in China (7.2 vs. 2.3%) [35]. The excess in COVID-19 mortality was higher in men than in women living in northern cities versus in central and southern Italy (men: +87% and +70% and women: + 17% and + 9%, respectively), with an increasing trend by age [36].

From May 4, a gradual reopening of financial and commercial activities has taken place (known as “Phase two” of the national sanitary emergency). A few, short-term studies have already shown the impact of lockdown on the mental health of the Italian general population in the first days of “Phase one” [37–39]. We have decided to carry out an online survey using several validated assessment instruments in order to evaluate the impact of the lockdown on the mental health of Italian population throughout the different weeks of Phase one [40]. In particular, in this paper we aim to: (a) report the levels of depressive, anxiety and stress symptoms in a large sample of the Italian general population; (b) explore the levels of depressive, anxiety and stress symptoms during the different weeks of lockdown; and (c) identify possible risk and protective factors for mental health outcome.

## Methods

### Study design

The COvid Mental hEalth Trial (COMET) is a national trial coordinated by the University of Campania “Luigi Vanvitelli” (Naples) in collaboration with nine university sites: Università Politecnica delle Marche (Ancona), University of Ferrara, University of Milan Bicocca, University of Milan “Statale”, University of Perugia, University of Pisa, Sapienza University of Rome, “Catholic” University of Rome, University of Trieste. The Center for Behavioral Sciences and Mental Health of the National Institute of Health in Rome has been involved in the study by supporting the dissemination and implementation of the project according to the clinical guidelines produced by the National Institute of Health for managing the effects of the COVID-19 pandemic.

The COMET trial includes three phases: phase one consists in the dissemination of a survey on the impact of lockdown and its related containment measures on the mental health of the Italian general population; the second phase consists in the development of a new psychosocial online supportive intervention [41–48] for the management of the consequences on mental health of the pandemic; the last phase consists in the evaluation of the efficacy and feasibility of the experimental psychosocial intervention in a randomized control trial. The results of phase 1 are described in this paper. The study has been approved by the Ethical Review Board of the coordinating center (protocol number: 0007593/i).

### Primary and secondary outcomes

The primary outcome of the study is the severity of depressive-anxiety symptoms evaluated with the Depression, Anxiety, Stress Scale (DASS-21) [49]. Secondary outcomes include the levels of global mental health status, of obsessive–compulsive and post-traumatic symptoms, presence and severity of insomnia, the levels of perceived loneliness and the presence of suicidal ideation/suicidal thoughts. Furthermore, exploratory variables include coping strategies, levels of post-traumatic growth, perceived social support and resilience.

### Assessment tools

The DASS-21 evaluates the general distress on a tripartite model of psychopathology [49] and is a reliable and valid measure in assessing mental health in the general population [50], which has been already adopted in previous research on SARS [51] and COVID-19 [14, 52]. The DASS consists of 21 items grouped in three subscales: depression, anxiety, and stress. Each item is rated on a 4-level Likert scale, from 0 (never) to 3 (almost always). The total score is calculated by adding together the response values of each item, with higher scores indicating more severe levels of depressive, anxiety, and stress symptoms. The score at the DASS—depression subscale (e.g., “I felt that I had nothing to look forward to”) is divided into normal (0–9), mild (10–12), moderate (13–20), severe (21–27), and extremely severe depression (28–42). The score at the DASS—anxiety subscale (e.g., “I was worried about situations in which I might panic and make a fool of myself”) is divided into normal (0–6), mild (7–9), moderate (10–14), severe (15–19), and extremely severe anxiety (20–42). The score at the DASS—stress subscale (e.g., “I tended to over-react to situations”) is divided into normal (0–10), mild (11–18), moderate (19–26), severe (27–34), and extremely severe stress (35–42).

The General Health Questionnaire (GHQ)—12 items version explores participants’ mental health status through six positively worded items (e.g., “Have you been able to concentrate”?) and six negatively worded items (e.g., “Have you lost much sleep over worry?”). The standard scoring method recommended by Goldberg for the need of case identification is called “GHQ method.” Scores for the first two types of answers are “0” (positive) and for the other two are “1” (negative). Threshold ≥ 4 at GHQ identifies people with a probability > 80% of having a mental health problem [53].

The Obsessive–Compulsive Inventory—Revised version (OCI-R) consists of 18 items rated on a 5-level Likert scale, ranging from 0 to 4. The total score is calculated by adding all single items. Scores above the threshold of 21 are indicative of an OCD diagnosis [54].

The Insomnia Severity Index (ISI) includes seven items rated on a 5-level Likert scale (from 0 to 4), with a total score ranges from 0 to 28 [55].

The Suicidal Ideation Attributes Scale (SIDAS) consists of five items assessing frequency, controllability, closeness to attempt, level of distress associated with suicidal thoughts and impact on daily functioning. Each item is assessed on a 10-level Likert scale, with a total score ranging from 0 to 50. In case of scoring “0—Never” to the first item, all other items are skipped, and the total score is zero. The presence of any suicidal ideation is considered indicative of risk for suicidal behavior, while a cut-off of 21 is used to indicate high risk of suicidal behavior [56].

The Severity-of-Acute-Stress-Symptoms-Adult scale (SASS), which consists of nine items rated on a 5-point scale (from 0 = Not at all to 4 = Extremely), has been used to assess the presence of traumatic stress symptoms. The total score ranges from 0 to 28, with higher scores indicating a greater severity of acute stress disorders [57].

The Impact of Event Scale (IES)—short version measures the traumatic reactions in people who have experienced traumatic events. Each item is rated on a 5-point scale ranging from 0 (not at all) to 5 (often). The IES evaluates the dimensions of intrusion, avoidance, and alteration in arousal [58].

The UCLA loneliness scale—short version is an eight-item scale designed to measure subjective feelings of loneliness, as well as feelings of social isolation. Each item is scored on a 4-level Likert scale from 0 = never to 3 = often [59].

The Brief-COPE consists of 28 items grouped in 14 subscales [60]. Each item is rated on a 4-level Likert scale from 0 = “I have not been doing this at all” to 3 = “I have been doing this a lot.” Coping strategies are divided in maladaptive strategies, including denial, venting, behavioral disengagement, self-blame, self-distraction and substance abuse, and adaptive coping strategies, which include emotional support, use of information, positive reframing, planning and acceptance. Two other subscales include religion and humour.

The short form of Post-Traumatic Growth Inventory (PTGI) is a 10-item assessment instrument grouped into five dimensions: relating to others, new possibilities, personal strengths, spiritual change, and appreciation of life. Items are rated on a 6-point Likert scale, from 0 = “I did not experience this change as a result of my crisis” to 5 = “I experienced this change to a very great degree as a result of my crisis”. Higher scores indicate higher levels of post-traumatic growth [61].

The Connor–Davidson resilience scale (CD-RISC), which includes 10 items rated on a 6-level Likert scale, is subdivided into the following five factors: (a) personal competence, high standards, and tenacity; (b) trust in one's instincts, tolerance of negative affect, and strengthening effects of stress; (c) positive acceptance of change and secure relationships; (d) control; and (e) spiritual influences. Higher values indicate higher levels of resilience [62].

The Multidimensional Scale of Perceived Social Support (MSPPS) consists of 12 items rated on a 7 level-Likert scale, from 1 = “absolutely false” to 7 = “absolutely true”. Items are grouped into three dimensions: family support, support by friends and support by significant others. Higher values correspond to higher levels of perceived support [63].

The Maslach Burnout Inventory (MBI) has been used to evaluate the levels of burn-out in medical personnel [64]. Data regarding healthcare professionals are not included in this paper since they are out of the aims of the study and will be reported in subsequent analyses.

Respondents’ socio-demographic (e.g., gender, age, geographical region, working and housing condition, etc.) and clinical information (e.g., having a previous physical or mental disorder, using illicit drugs or medications, etc.) have been collected through an ad-hoc schedule.

### Methodology

The phase one of the COMET trial consists in an online survey carried out between March and May 2020 in the Italian adult population. The survey has been implemented through a multistep procedure: (a) email invitation to healthcare professionals and their patients; (b) social media channels (Facebook, Twitter, Instagram); (c) mailing lists of universities, national medical associations and associations of stakeholders (e.g., associations of users/carers); and (d) other official websites (e.g., healthcare or welfare authorities websites).

The online survey has been set up through EUSurvey, a web platform promoted by the European Commission (2013). The survey has been officially launched on March 30, 2020, and it takes approximately 30 min (range 15–45 min) to be completed.

The full study protocol is available elsewhere [40].

### Statistical analysis

Descriptive statistics were performed in order to describe the socio-demographic and clinical characteristics of the sample. The time points of data collection were recorded and coded using the variable “Week” (categorical: Week 1—March 30/April 8 (reference category); Week 2—April 9/April 15; Week 3—April 16/April 22; Week 4—April 23/April 29; Week 5—April 30/May 4). According to the official data of the Italian Ministry of Health, Lombardy, Piedmont, Veneto and Emilia-Romagna were the regions with the highest rate of new COVID cases and of COVID-related mortality (http://www.salute.gov.it/portale/nuovocoronavirus). Therefore, geographical regions of respondents were recoded using a binary variable “Severely impacted area.” This variable has been entered in the regression model in order to evaluate the direct impact of living in an area with a higher risk of being infected rather than the impact of geographical area *per se*. We hypothesized that individuals living in the most affected areas should have presented more severe symptoms compared with those living in less affected areas.

By order of the Italian health authority, persons subject to quarantine are forbidden to move from home or residence for 14 days, with the aim to separate persons exposed (or potentially exposed) to the infectious agent from the general community for reducing the contagion rate. People who have been subjected to those restrictions were coded using the binary variable “Quarantine.”

In order to adjust for the likelihood of participants of being exposed to COVID infection in each week, a propensity score was calculated [65]. This methodological choice was due to the fact that the propensity score produces a better adjustment for differences at baseline, rather than simply including potential confounders in the multivariable models. The propensity score was calculated using as independent variables age, gender, socioeconomic status and living in a severely impacted area [66]. In the final regression model, the inverse probability weights, based on the propensity score, were applied in order to model for the independence between exposure to the infection, outcomes and estimation of causal effects.

In order to evaluate factors associated with the severity of depressive, anxiety and stress symptoms at DASS-21 (primary outcomes), multivariate linear regression models were performed, including as independent variables: being infected by COVID-19, having a pre-existing mental disorder, being a healthcare professional. Furthermore, in order to evaluate the impact of the duration of lockdown and of other related containment measures on the primary outcomes, the categorical variable “Week” was also entered in the regression models. The models were adjusted for the rate of new COVID cases and of COVID-related mortality during the study period, as well as for several socio-demographic characteristics, such as gender, age, occupational status, having a physical comorbid condition, hours spent on Internet, levels of perceived loneliness, health status, number of cohabiting people, level of satisfaction with one’s own life, with cohabiting people, with the housing condition. Missing data have been handled using the multiple imputation approach [67].

Statistical analyses were performed using the Statistical Package for Social Sciences (SPSS), version 17.0 and STATA, version 15. For all analyses, the level of statistical significance was set at *p* < 0.05.

## Results

### Sociodemographic characteristics

The final sample consisted of 20,720 participants, 71% female (*N* = 14,720), with a mean age of 40.4 (14.3) years; half of respondents were in a stable relationship, living with the partner (52.2%, *N* = 10,808) (Table 1). The vast majority of participants were employed (70.1%, *N* = 14,518) and 34.2% (*N* = 7,089) shifted to smart working during the pandemic. (*N* = 1,302) of respondents lost their job during the pandemic. 80% spent more time on Internet than usual, more frequently for instant messaging (85.3%, *N* = 17,683), searching for information (81.6%, *N* = 16,899), or using social networks (62.1%, *N* = 12,867). About 14.5% of cases (*N* = 3,012) suffered from a pre-existing physical illness, mainly cardiovascular diseases (24.7%), osteo-articular disorders (17.5%), thyroid dysfunctions (9%), and diabetes/dyslipidaemia (7.6%). 5.5% (*N* = 1,133) reported to have a pre-existing mental disorder, more frequently anxiety (34.3%) and depressive disorders (35.5%). 14% of respondents (*N* = 2,907) were healthcare professionals.

**Table 1.** Socio-demographic and clinical characteristics of the sample (*N* = 20,720).

Age, years, mean ± SD	40.4 ± 14.3
Age groups, % (*N*)	
18–24 years old	15.2 (3,151)
25–55 years old	65.2 (13,514)
55–64 years old	14.0 (2,904)
over 65 years old	5.6 (1,151)
Gender, *F*, % (*N*)	71 (14,720)
Living with partner, yes, % (*N*)	52.2 (10,808)
University degree, yes, % (*N*)	62 (12,844)
Employed, yes, % (*N*)	70 (14,518)
Lost job due to the pandemic, yes, % (*N*)	6.3 (1,302)
Are you practicing smart working, yes, % (*N*)	34.2 (7,089)
Spending more time on Internet, yes, % (*N*)	80.1 (16,598)
Any comorbid physical condition(s), yes, % (*N*)	14.5 (3,012)
Any mental health problem(s), yes, % (*N*)	5.5 (1,133)
Have you been infected by COVID-19, yes, % (*N*)	1.4 (296)
Have you been isolated due to COVID-19 infection, yes, % (N)	1.5 (316)
Have you been in contact with someone affected by COVID-19, % (*N*)	4.2 (866)
Clinical characteristics	
General Health Questionnaire—global score, mean ± SD (range: 0–12)	5.6 ± 1.6
Obsessive compulsive inventory—global score, mean ± SD (range: 0–72)	10.7 ± 8.2
Insomnia severity index, mean ± SD (range: 0–28)	9.8 ± 5.2
Suicidal Ideation Attributes Scale (SIDAS), mean ± SD (range: 0–50)	4.9 ± 6.6
Severity of Acute Stress Symptoms- Adult, mean ± SD (range: 0–28)	6.0 ±4 .9
Impact of event scale, mean ± SD (range: 0–5)	
Intrusion	1.1 ± 1.9
Avoidance	2.3 ± 2.0
Hyperarousal	2.5 ± 1.9
Loneliness, mean ± SD (range: 0–24)	19.1 ± 3.6
Coping strategies, mean ± SD (range: 1–4)	
Maladaptive strategies	
Self-distraction	2.7 ± 0.8
Denial	1.5 ± 0.7
Venting	2.7 ± 0.8
Behavioral disengagement	1.6 ± 0.6
Self-blame	2.4 ± 0.8
Substance use	1.2 ± 0.5
Adaptive strategies	
Acceptance	3.1 ± 0.7
Active	2.9 ± 0.8
Emotional support	2.4 ± 0.8
Use of information	2.4 ± 0.8
Positive reframing	2.3 ± 0.7
Planning	3.0 ± 0.8
Other	
Religion	1.9 ± 0.9
Humour	2.1 ± 0.8
Post-traumatic growth inventory, mean ± SD (range: 0–10)	
Personal strength	2.1 ± 3.4
Spiritual change	3.7 ± 2.9
Appreciation for life	6.4 ± 3.2
Relating to others	5.3 ± 1.6
New possibilities	5.8 ± 1.6
Connor–Davidson Resilience Scale, mean ± SD (range: 0–40)	31.3 ± 10.4
Multidimensional scale of perceived social support, mean ± SD (range: 4–28)	
Family support	21.1 ± 6.7
Friends support	20.3 ± 6.5
Support from other relevant ones	22.3 ± 6.7

Abbreviations: COVID-19, Coronavirus disease 2019; SD, Standard deviation.

### Clinical characteristics

Almost all participants (91.2%, *N* = 18,882) scored above the threshold of 4 at the GHQ, indicating the risk of having any mental health problem. In particular, depressive symptoms were moderate in 36.5% of respondents (*N* = 10,124) and severe or extremely severe in 12.4% (N = 2,555); anxiety symptoms were moderate in 16.7% (*N* = 3,469) of respondents and severe or extremely severe in 17.6% (*N* = 3,633); stress symptoms were at least moderate in 41.6% (*N* = 8,619) (Table 2).

**Table 2.** Levels of depression, anxiety and stress during the lockdown period.

DASS—Depression subscale, mean ± SD: 12.2 ± 7.5		
	Global sample	
	N	%
Normal (range: 0–9)	7,608	36.7
Mild (range: 10–12)	2,980	14.4
Moderate (range: 13–20)	7,569	36.5
Severe (range: 21–27)	2,271	11.0
Extremely severe (range: 28–42)	284	1.4
DASS—Anxiety subscale, mean ± SD: 7.4 ± 6.8		
	Global sample	
	N	%
Normal (range: 0–6)	10,836	52.3
Mild (range: 7–9)	2,773	13.4
Moderate (range: 10–14)	3,469	16.7
Severe (range: 15–19)	2,085	10.1
Extremely severe (range: 20–42)	1,548	7.5
DASS—Stress subscale, mean ± SD: 16.3 ± 7.1		
	Global sample	
	N	%
Normal (range: 0–10)	3,877	18.7
Mild (range: 11–18)	8,216	39.7
Moderate (range: 19–26)	7,728	37.3
Severe (range: 27–34)	891	4.3
Extremely severe (range: 35–42)	0	0

Abbreviations: DASS, Depression, Anxiety, Stress Scale; SD, Standard deviation.

Moderate to severe levels of insomnia were found in 38.8% of respondents (*N* = 8,031). About 11.3% (*N* = 2,332) of the sample scored above the threshold for clinical relevance of obsessive-compulsive symptomatology, with a global severity of obsessive–compulsive symptoms of 10.7 (± 8.2) at OCI-R. Suicidal ideation is reported by 14.2% (*N* = 2,976) of the sample, with a mean score of 4.9 (6.6) at the SIDAS.

Participants showed high levels of avoidance and hyperarousal symptoms (2.3 ± 2.0 and 2.5 ± 1.9, respectively), with lower levels of intrusive symptoms (1.1 ± 1.9) at the IES-R. 17.2% (*N* = 3,558) reported to feel alone, 29.4% (*N* = 6,080) to feel excluded by others and 36.9% (*N* = 7,646) feel that “other people are around them, but not together with them”, at the UCLA.

At the Brief-COPE, we found that respondents more frequently used adaptive coping strategies, such as planning (38.9% of participants, *N* = 8,059), acceptance (44.2%, *N* = 9,156), and active coping (36.2%, *N* = 7,503). As regards maladaptive coping strategies, 10.2% (*N* = 2,106) of the sample used venting, 16% (*N* = 3,321) self-blame and 26.2% (*N* = 5,429) self-distraction. Moreover, a relatively high proportion of respondents (18.4%; *N* = 3,777) reported to use psychoactive medications in order to cope with the situation.

At the PTGI, participants reported that they found “something positive” out of this situation, with high levels of “appreciation for life” (51.3%, *N* = 10,625), feeling closer to other people (40.5%, *N* = 8,388), being more satisfied of everyday life (42.1%, *N* = 8,728) and increased ability to handle difficult situations (43.9%, *N* = 9,093). Furthermore, respondents reported a good level of resilience with a mean score of 31.3 ± 10.4 at the CD-RISC.

Finally, the majority of participants declared to feel supported by family (70.6%, *N* = 14,623) and friends (69.8%, *N* = 14,461), with a mean score of 21.1 ± 6.7 at the MSPPS family support subscale and of 20.3 ± 6.5 at the MSPPS friend support subscale (Table 1).

### Variations in the levels of depressive, anxiety and stress symptoms over time

**Figure 1.**
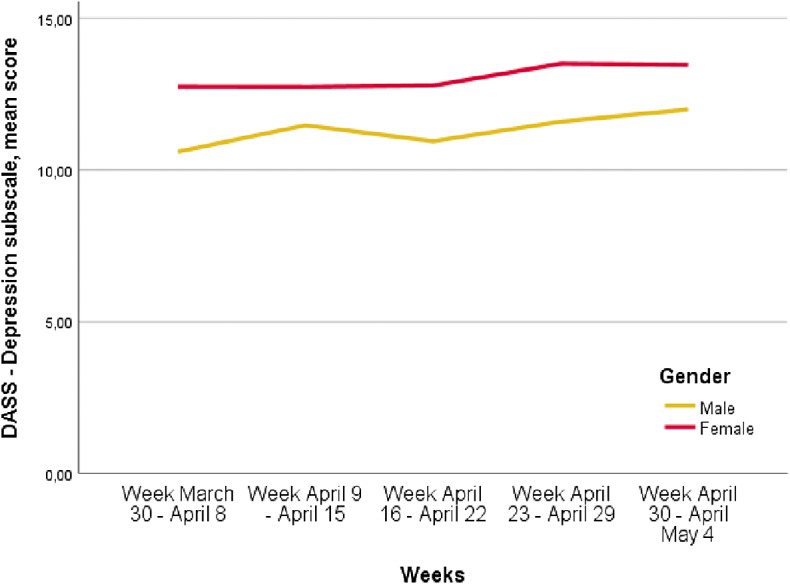
Depression, Anxiety, Stress Scale (DASS)—Depression mean score variation over time, *p* < 0.0001 (*p*<0.0001 refers to the differences of the different time points).

**Figure 2.**
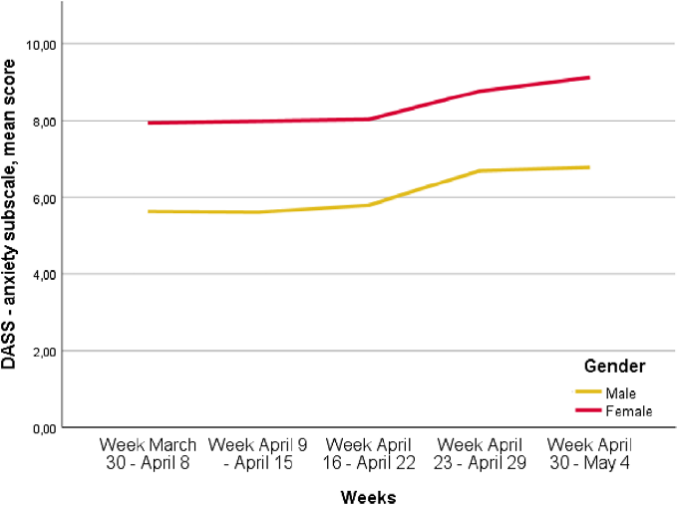
Trend of Depression, Anxiety, Stress Scale (DASS)—Anxiety mean scores over time in men and women (*p* < 0.0001 refers to the different time points).

**Figure 3.**
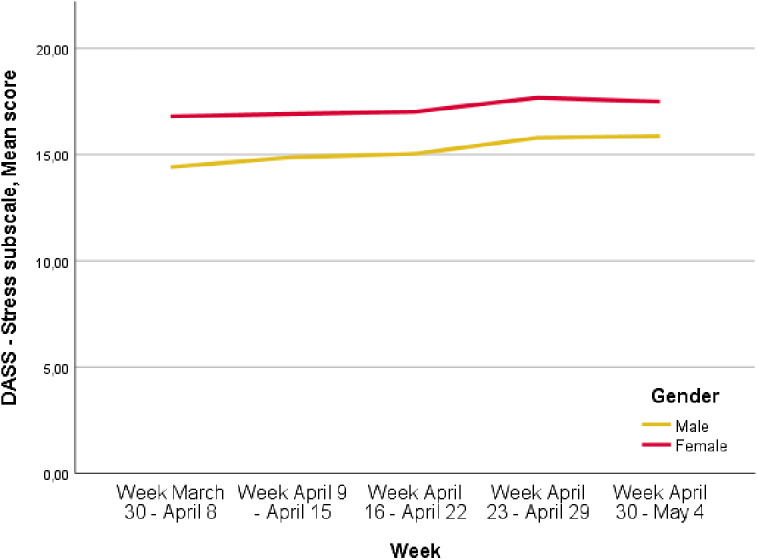
Depression, Anxiety, Stress Scale (DASS)—Stress mean score variation over time, *p* < 0.0001 (*p* < 0.0001 refers to the differences at the different time points).

The levels of depressive symptoms increased over the period of the lockdown. In particular, depressive symptoms changed from 12.1 *±* 7.5 in the week March 30 to April 8 to 13.1 *±* 7.4 in the week April 30 to May 4 (*p* < 0.0001). Anxiety symptoms increased from 7.5 ± 6.7 in the week March 30 to April 8 to 8.5 ± 7.2 in the week April 30 to May 4 (*p* < 0.0001). Furthermore, the levels of stress symptoms increased from 16.0 ± 7.2 in the week March 30 to April 8 to 17.2 *±* 6.7 in the week April 30 to May 4 (*p* < 0.0001). These increases were higher in female participants compared to males (Figures 1–3; *p* < 0.0001).

### Factors associated with depressive, anxiety and stress symptoms

The multivariate regression analyses are reported in Table 3. According to the multivariate regression models, weighted for the propensity score, weeks of exposure to the pandemic and to the related containment measures were significantly associated with worsening of depressive symptoms, with Beta coefficient ranging from 0.4 (95% Confidence Interval, CI: 0.1–0.8) during the week April 9–15 to 1.5 (95% CI: 0.8–2.4) in the week April 30–May 4. Similarly, also anxiety symptoms (from Beta: 0.4, 95% CI: 0.1–0.7 in the week April 9–15 to Beta: 2.4, 95% CI: 1.7–3.1 in the week April 30–May 4) and stress symptoms (Week April 30–May 4, Beta: 1.5, 95% CI: 0.7–2.3; Week April 16–22, Beta: 0.8, 95% CI: 0.3–1.3) tended to increase over time, even after controlling for the potential role of confounders, such as infection rate and mortality rate for COVID-19 in Italy.

**Table 3.** Regression models weighted by propensity score.

	DASS—Depression				DASS—Anxiety				DASS—Stress			
	Beta coefficient	*p* value	95% Confidence interval		Beta coefficient	*p* value	95% Confidence interval		Beta coefficient	*p* value	95% Confidence interval	
			Lower bound	Upper bound			Lower bound	Upper bound			Lower bound	Upper bound
Intercept	7.816	0.000	6.294	9.338	4.531	0.000	3.161	5.9	12.318	0.000	10.851	13.785
Quarantine, yes	**0.988**	**0.014**	0.197	1.780	−0.157	0.666	−0.869	0.555	−0.147	0.706	−0.910	0.616
Having a pre-existing mental health problem	**3.475**	**0.000**	2.670	4.279	**4.224**	**0.000**	3.500	4.948	0.449	0.257	−0.327	1.224
Having been infected by COVID	**1.284**	**0.000**	0.604	1.963	**1.435**	**0.000**	0.823	2.047	0.136	0.684	−0.519	0.791
Being healthcare professional	−0.064	0.867	−0.815	0.687	0.284	0.411	−0.393	0.960	0.223	0.547	−0.502	0.947
Having a pre-existing physical comorbid condition	**0.859**	**0.000**	0.568	1.150	**1.370**	**0.000**	1.108	1.632	**0.718**	**0.000**	0.437	0.998
Gender female, ref. Male	**1.885**	**0.000**	1.6	2.103	**2.177**	**0.000**	1.981	2.372	**2.148**	**0.000**	1.939	2.358
Occupational status, ref. employed												
housewife	**1.118**	**0.000**	0.523	1.713	**1.503**	**0.000**	0.967	2.038	0.250	0.393	−0.324	0.823
unemployed	**1.233**	**0.000**	0.866	1.599	**0.624**	**0.000**	0.294	0.953	−**0.709**	**0.000**	−1.062	−0.356
retired	**0.872**	**0.000**	0.617	1.127	**0.327**	**0.005**	0.098	0.556	−**0.415**	**0.001**	−0.660	−0.170
Being in one of the most affected Italian regions	**0.264**	**0.017**	0.048	0.481	**0.460**	**0.000**	0.265	0.654	−0.092	0.389	−0.300	0.117
Time to exposure, ref. week March 30 to April 8												
Week April 15 to April 9	**0.462**	**0.008**	0.119	0.806	**0.413**	**0.009**	0.104	0.723	**0.329**	**0.050**	0.002	0.661
Week April 16 to April 22	**0.792**	**0.003**	0.274	1.309	**1.022**	**0.000**	0.557	1.488	**0.835**	**0.001**	0.336	1.334
Week April 23 to April 29	**1.467**	**0.000**	0.869	2.064	**1.841**	**0.000**	1.304	2.379	**1.424**	**0.000**	0.848	2.000
Week April 30 to May 4	**1.581**	**0.000**	0.786	2.377	**2.372**	**0.000**	1.656	3.088	**1.515**	**0.000**	0.748	2.282
Age	**−0.042**	**0.000**	−0.050	−0.034	**−0.046**	**0.000**	−0.053	−0.039	−0.**051**	**0.000**	−0.059	−0.043
Number of cohabiting people	**−0.188**	**0.000**	−0.263	−0.114	−0.012	0.736	−0.079	0.056	**0.214**	**0.000**	0.142	0.285
Global satisfaction with life	**−0.430**	**0.000**	−0.479	−0.381	−**0.228**	**0.000**	−0.272	−0.184	−**0.198**	**0.000**	−0.246	−0.151
Satisfaction with cohabiting people	**−0.054**	**0.026**	−0.101	−0.006	−**0.138**	**0.000**	−0.180	−0.095	0.039	0.096	−0.007	0.084
Satisfaction with housing	0.003	0.901	−0.045	−0.051	−0.043	0.051	−0.085	0.000	0.059	0.012	0.013	0.104
GHQ—global score	**0.367**	**0.000**	0.335	0.398	**0.219**	**0.000**	0.190	0.247	**0.308**	**0.000**	0.278	0.338
Hours spent on Internet	**0.151**	**0.000**	0.116	0.186	**0.178**	**0.000**	0.146	0.210	**0.055**	**0.002**	0.021	0.089
Loneliness global score	**0.084**	**0.000**	0.054	0.114	**0.045**	**0.001**	0.018	0.072	0.029	0.053	0.000	0.057
Cases of COVID in Italy	0.001	0.045	4.96	0.000	0.001	0.004	9.0	0.000	0.000	0.002	0.000	0.001
Mortality rate of COVID in Italy	0.001	0.379	−0.001	0.002	0.002	0.015	0.000	0.003	0.000	0.541	−0.002	0.001

Abbreviations: COVID, Coronavirus disease; GHQ, General Health Questionnaire.

Bold characters indicate significant value.

Other factors associated with worse levels of depressive-anxiety symptoms include being affected by a pre-existing mental disorder (DASS–Depression: Beta: 3.5, 95% CI: 2.7–4.3; DASS–Anxiety: Beta: 4.2, 95% CI: 3.5–4.9), having been infected by the COVID-19 (DASS-Depression: Beta: 1.3, 95% CI: 0.6–1.9; DASS–Anxiety: Beta: 1.4, 95% CI: 0.8–2.0), and having a pre-existing physical disease (DASS—Depression: Beta: 0.9, 95% CI: 0.6–1.1; DASS–Anxiety: Beta:1.3, 95% CI: 1.1–1.6; DASS–Stress: Beta: 0.7, 95% CI: 0.4–0.9). Moreover, the risk of severe depressive, anxiety and stress symptoms were double in female compared to male participants (*p* < 0.05).

Protective factors against the development of psychiatric symptoms included higher levels of satisfaction with one’s own life and with cohabiting people, and living with a higher number of family members (*p* < 0.05).

## Discussion

The COMET is the first trial evaluating the global impact of the COVID-19 pandemic and its related containment measures on several dimensions of mental health in a large sample of the Italian population.

One of our main findings is the presence of moderate to severe levels of depressive, anxiety, and stress symptoms which are higher than those found in China [52, 68, 69]. This difference could be due to the type of immediate health response in the two countries, with clear lockdown measures from the beginning of the pandemic in China [70] and a more fragmented preventive approach in Italy, which may have increased the levels of fears and uncertainty in this country [37–39, 71]. In fact, the uncertainties about the pandemic progression, the “hypochondriac concerns” [72] and fear that the epidemic is difficult to control represent triggering factors for the development of mental health problems [73, 74]. Moreover, studies carried out during natural disasters, war, fires and terroristic attacks found high levels of depressive/anxiety-related symptoms in the general population [75–81], but nevertheless they were significantly lower compared to those we found in our study. These data confirm that the current pandemic is an unprecedented event in terms of its impact on the mental health of the general population.

A second interesting finding of our survey is that the levels of anxiety, depressive and stress symptoms increased over time, being more severe in the last weeks of the lockdown, as also found in our regression models controlled for all socio-demographic characteristics of respondents. This finding confirms the hypothesis that the duration of containment measures significantly influences mental health and well-being of the general population, as also found by Sibley et al. [73] in a sample of the general population in New Zealand. Moreover, this trend has not been influenced by the rate of COVID cases and COVID mortality rates in Italy, highlighting that these public measures—although being necessary for infection control—should be removed as soon as possible in order to safeguard public mental health.

Female participants are at higher risk of developing depressive-anxiety symptoms, as already shown in small Italian samples [82, 83] and in previous outbreaks [84]. This finding can be due to the higher incidence in women of anxiety-depressive disorders [85–88] and of anxious, cyclothymic and depressive temperaments in women [37], also in community-based samples [89].

Moreover, being affected by a pre-existing mental health problem represents an independent significant risk factor for the development of depressive, anxiety and stress symptoms, as already reported by Plunkett et al. [90] and Hao et al. [14]. This finding suggests the need to provide as soon as possible adequate and tailored supportive interventions to mentally ill patients, who represent fragile and at-risk individuals that have been overlooked during the initial phases of the pandemic [91–95].

During the lockdown participants reported an increased time spent on Internet, which was associated with a higher risk of developing mental health problems, thus not confirming our hypothesis of a protective effect played by Internet on mental health. This finding may be due to the diffusion through Internet of uncontrolled and unreliable information and fake news, which may have increased the levels of anxiety and depressive symptoms in people who are alone and with lower levels of education [96]. This finding highlights the need for media professionals to receive an appropriate training, in order to provide unbiased and non-sensationalistic information during catastrophic events.

Being unemployed, retired or housewife was significantly associated with higher levels of anxiety-depressive symptoms [13]. In the UK, belonging to a socio-economic disadvantaged group increased the risk of developing mental health problems, according to a gradient across the different weeks of the lockdown [17]. This finding highlights the need for global, multi-level socio-economic initiatives aiming to reduce the negative effect of the pandemic on the society [97]. These data should also be interpreted considering the high rate (14.5%) of suicidal ideation/suicidal thoughts found in our sample. The rate of suicidal ideation found in our sample is quite impressive, compared with the 3% found in a previous epidemiological study carried out in Italy [98]. Several factors may contribute to the increased rate of suicidal ideation in the Italian general population, including uncertainty about the future, loneliness, physical distancing, unemployment, economic recession and interpersonal violence [99]. All these risk factors should be taken into account in the implementation of actions aiming to prevent suicide [100–102].

Participants reported several disturbances in sleep quality and patterns, as already found in other studies carried out in China and in other European countries [103,104]. The public health containment measures implemented worldwide have markedly changed daily routines and may have had an impact on sleep pattern and on the risk of developing other mental health problems [105,106]. In order to develop tailored innovative preventive and/or therapeutic strategies, the specific socio-demographic and clinical predictors of sleep problems should be identified.

Finally, good levels of perceived social support and of post-traumatic growth in the aftermath of the pandemic have been reported from the Italian general population participating in our survey. It may be that the Italian socio-cultural context, with strong family ties and social relationships, may have positively impacted on the perception of mutual social support [107]. However, longitudinal studies may help to evaluate changes in the levels of post-traumatic growth, resilience, and social support in the subsequent phases of the ongoing health crisis [108].

Our study has several strengths. This is the first study carried out in different geographic Italian regions with a large sample from the general population during the lockdown period. Validated and reliable assessment instruments have been used in order to investigate several domains of mental health and psychological well-being according to a propensity score analysis. Moreover, as primary outcome we have selected the same assessment tool (the DASS-21) used in studies carried out in China in order to allow direct comparisons between the two countries. Although the DASS-21 scores in the Italian general population prior of the pandemic are not available, the comparison of our findings with national statistics (https://www.epicentro.iss.it/mentale/epidemiologia-italia) document higher levels of anxiety, depressive and stress symptoms during the pandemic. Therefore, the increased frequency of depressive-anxiety symptoms in our sample could be interpreted as COVID-19 related, although this causal association should be further investigated. In any case, we believe that the analysis of DASS-21 over the different weeks of lockdown provide an important contribution to the field in order to clarify the direct impact of the pandemic on the mental health.

We are aware that the use of an online tool is not the best methodological choice, since it may have excluded elderly people or those living in socially disadvantaged contexts [109]. However, this choice was necessary in order to reach a large portion of Italian population in a short time and in a pandemic situation, when face-to-face contacts are forbidden [110].

Finally, it must be acknowledged that collected data are related to depressive or anxiety symptoms, which cannot be considered as sufficient to formulate a diagnosis of depressive/anxiety disorders. Therefore, this survey represents an initial step for the promotion of appropriate screening procedures in the general population for the early detection of full-blown mental disorders.

The present study has several clinical implications: (a) to promote mass screening campaigns for the general population in order to identify the presence of subthreshold mental disorders; (b) to disseminate informative intervention on how to deal with the mental health consequences of the pandemic; and (c) to support at-risk population—mainly people with pre-existing mental health problems and COVID-19 patients—with tailored innovative psychosocial interventions.

In conclusion, there is the need to address mental health needs as an integral part of COVID-19 response. In fact, although physical isolation and lockdown represent essential public health measures for containing the spread of the COVID-19 pandemic, they are a serious threat for mental health and well-being of the general population. It is necessary to get prepared if a next emergency will come, in order to provide appropriate community-based mental health service responses to the population.

## Data Availability

The dataset is not available for sharing.
